# A cross-sectional study examining predictors of visit frequency to local green space and the impact this has on physical activity levels

**DOI:** 10.1186/s12889-016-3050-9

**Published:** 2016-05-20

**Authors:** Elliott P. Flowers, Paul Freeman, Valerie F. Gladwell

**Affiliations:** School of Biological Sciences, University of Essex, Colchester, CO4 3SQ UK

**Keywords:** Local green space, Nature relatedness, Perceptions, Physical activity, Green exercise

## Abstract

**Background:**

Lack of physical activity (PA) is a growing public health concern. There is a growing body of literature that suggests a positive relationship may exist between the amount of local green space near one’s home and PA levels. For instance, park proximity has been shown to predict PA levels amongst certain populations. However, there is little evidence for the role of relatedness towards nature and perceptions of local green space on this relationship. The aim of this study was to examine, in a National UK sample, whether subjective indices associated with local green space were better predictors of visit frequency to local green space and PA levels compared to objectively measured quantity of local green space.

**Methods:**

A cross-sectional survey was designed. From a random sample, 2079 working age adults responded to an online survey in September 2011. Demographics, self-reported PA, objective measures of the local environment (including local green space, road coverage, and environmental deprivation), were assessed in conjunction with perceptions of local green space and nature relatedness. Quantity of local green space was assessed by cross-referencing respondents’ home postcodes with general land use databases. Regression models were conducted to assess which of our independent variables best predicted visit frequency to local green space and/or meeting PA guidelines. In addition, an ordinal regression was run to examine the relationship between visit frequency to local green space and the likelihood of meeting national PA guidelines.

**Results:**

Nature relatedness was the strongest predictor for both visit frequency to local green space and meeting PA guidelines. Results show that perceived quality is a better predictor of visit frequency to local green space than objective quantity of local green space. The odds of achieving the recommended amount of PA was over four times greater for people who visited local green space once per week compared to never going (OR 4.151; 95 % CI, 2.40 to 7.17).

**Conclusions:**

These results suggest that perceptions of local green space and nature relatedness play an important role in the relationship between local green space and PA. Considering the known health benefits of PA, our results are potentially important for public health interventions, policy making and environmental planning.

## Background

Physical activity (PA) is a well-known contributor to good health [1]. Current guidelines for aerobic activity recommend that adults should spend at least 150 min per week in moderately intensive PA or 75 min of vigorous PA (or any combination of the two) [2, 3]. In the most recent Health Survey for England, unfortunately, only 67 % of men and 55 % woman met the recommended guidelines [4]. This is replicated to a lesser extent throughout the whole world, with 20 % of men and 27 % of women considered not to meeting the guidelines [5]. Therefore, increasing levels of PA is a major priority in Public Health, particularly in Westernised countries [6].

An increasing number of studies have investigated the impact of the natural environment on PA behaviours and health. Systematic reviews have found that there is a positive correlation between the availability of local green space (LGS) and PA levels [7–9]. The mechanisms for this, however, remain unclear.

Recently, a schematic model of the motivational processes underlying the relationship between natural environments and PA was proposed [10]. In a compelling argument, the authors suggested two distinct motivational pathways for visiting LGS: firstly the active use of natural environment and secondly as a contributor to active living. Consistent with these pathways, this study explores how feelings about nature influence visits to LGS (active use) and the subsequent relationship this has on PA (active living). Specifically, we examine the influence of both objective and subjective measures of the local environment on visit frequency to LGS and the likelihood of meeting PA guidelines. We also explore the influence of visit frequency to LGS on PA levels.

The literature in this area has predominately focused on two examples of green spaces, namely natural environments and urban green spaces, or a combination of the two [11]. By definition, natural environments are those that occur naturally on earth. They differ from urban green spaces in that they have had minimum human input in their design, creation, and maintenance [11]. Both are used as locations for recreational activities in modern society. For the purposes of this study, LGS is a combination of urban green space and natural environments in close proximity to the home.

Many recreational activities that take place in LGS involve some form of PA such as walking, jogging, and play [12–14]. Even less intense activities like photography, reading, and fishing often require individuals to walk to desired locations. Thus, visit frequency to LGS may be positively associated with overall PA levels and subsequently the likelihood of meeting PA guidelines.

A recent review of the impact of LGS on PA found that there is a huge variety of research methods employed within the field, including objective and subjective measures of LGS [10]. Studies using objective measures have predominately focused on specific locations and used a Geographical Information System (GIS) to assess LGS [15–17]. Using GIS, researchers can analyse geographical data and categorise into various land uses (domestic buildings, roads, green space etc.). Conversely, subjective measures embrace self-report questionnaires to provide vital insight into individuals’ perceptions of LGS (pLGS). Requiring fewer resources, subjective measures enable investigation of some variables over much larger geographic areas [18, 19]. Interestingly, when both objective and subjective measures have been used to determine quantity of LGS in the same geographical area contemporaneously, discrepancies have been found between perceptions of park proximity and actual distance to park [20] as well as perceived versus actual quantity [21].

To date, only a small number of studies have investigated the relationships between objectively measured LGS, PA, and health on a national scale [22–25]. These studies have found mixed results. For example, in the Netherlands, the quantity of LGS within a one km radius of home address was associated with 15 indicators of well-being [22]. In contrast, neighbourhood park access was not associated with body mass index (BMI) in New Zealand, although beach access was related to BMI [25].

Furthermore, two studies in the UK have also produced mixed results. In England, individuals who lived in the greenest quintile of England were 1.27 times (95 % CI, 1.13 to 1.44) more likely to meet PA guidelines than individuals in the least green quintile [23]. In contrast, no association was found between LGS and meeting PA guidelines in Scotland [25].

A systematic review [10] suggested that perceptions or subjective measures of LGS access are stronger predictors of PA than environmental barriers such as actual proximity (e.g., [18, 26]). For example, perceived access to LGS has been linked with PA levels in Canada [20] and Australia [27]. In the UK, a few localised studies have investigated the relationship between pLGS and green space usage (e.g., [19, 28]). Results from Oxford and Bristol found that the majority of people were satisfied with accessibility to LGS. In Bristol, however, despite good perceived access, only 31 % of participants visited LGS on a weekly basis. This suggests that other factors are likely to play a crucial role in the actual use of LGS and in turn PA levels.

Beyond the role of perceived access, it is important to consider the perceived quality of LGS. Commonly reported as ‘satisfaction with neighbourhood parks’, evidence suggests perceived quality of LGS is positively related to PA [18, 29]. This further highlights the potential importance of pLGS for PA.

In addition to perceptions of quality and access, individuals’ self-reported relationship with nature may be a crucial determinant of whether they engage in PA in LGS (termed Green Exercise). Evidence from recent studies suggests that nature relatedness (individual levels of connectedness with the natural world; NR) plays an important role in engagement with nature and subsequent benefits [30, 31]. Indeed, NR has been shown to predict travel distance to parks [30], time spent in gardens [30], and psychological well-being [32]. In their schematic model, Calogiuri et al. [10] proposed that feelings about nature influence intentions to visiting LGS.

In summary, there are a number of factors that influence the relationship between LGS and PA, including actual visits to the LGS. Objective (GIS measured quantity of LGS) and subjective (perceived access and quality) have been shown to predict visit frequency to LGS and overall PA. It is vital, however, to take a more nuanced approach to understand the role of perceptions in the relationship between LGS and PA. No study has investigated which pLGS have greatest impact on visit frequency to LGS and subsequent PA. The aims of the study, therefore, were to examine: 1) which objective and perceptual indices of LGS predict visit frequency to LGS? 2) which objective and perceptual indices of LGS predict whether participants meet PA guidelines? and 3) if visit frequency to LGS predicts whether participants meet PA guidelines? It was hypothesised that perceived access and quality of LGS and NR would be stronger predictors of visit frequency to LGS and PA than objectively measured LGS. It was also hypothesised that the likelihood of meeting PA guidelines will increase in a dose-response pattern with visit frequency to LGS.

## Method

The data used in the present study were extrapolated from a larger research project examining the effects of the environment and exercise on psychological health. Part of the project was conducted using an online questionnaire administered to participants in the 150,000 person Harris Poll panel of Great Britain. The research was approved by the University of Essex Research Ethics Committee and participants provided informed consent. Participants were selected at random from the base sample and invited by email to take part in the survey (*n* = 22,950). Data from the responding sample were collected over a 2 week period in late September 2011. Data collection was closed after 2 weeks as it reached the requested number of respondents.

This process yielded a sample of 2079 working age adults. In the current study, data were available for 1988 working age adults (997 males) ranging from 22 to 65 years (M = 43.19, SD = 11.46), which is the higher than the UK median of 39 years [33]. Only employed individuals were selected for this research in order to control for the impact of active commuting on visiting LGS and PA levels; 69.8 % were in full-time employment, 18.1 % were in part-time employment, and 12.2 % were self-employed.

The UK Meteorological Office [34] reported that in July and August 2011, mean temperatures were 0.5 to 1.0 °C below average across most of the UK. In contrast, during September, 2011 – during data collection – the mean temperatures were around 1.1 °C above average, making it the sixth warmest September in 100 years. Throughout September, most of England experienced below average rainfall; some parts of Northern England and Scotland, however, received over 50 % more rainfall than average [34].

Self-reported health was assessed with a single item which asked “How would you rate your health in the last month?” Participants responded on a Likert scale from “1 = Terrible” to “7 Excellent”. This was included as a covariate in all statistical analyses alongside age and gender.

Objective representation of the local environment was given as % of LGS available near home. This was calculated to ward level (primary unit of electoral geography), using participants’ home postcodes and Geoconvert (an online geography matching and conversion tool) [35]. For % of LGS, ward coded data were then entered into a database, available from CRESH.org.uk, which has previously been described [36]. In brief, the database used general land use across England, supplemented with a second database covering Scotland, Northern Ireland, and Wales and the coordination of information placed on the environment database [37]. The database provided specific % of LGS, including all vegetated areas larger than 5 m^2^ in area (excluding domestic gardens) for each ward in the UK. Green spaces included ranged from transport verges (narrow strip of land between carriageway and road boundary) and neighbourhood greens, to parks, playing fields and woodlands.

Perceived access to LGS was assessed by asking participants “How easy is it to get to the green space local to your home?” Participants responded from 1 = “Very difficult” to 7 = “Very easy”. Perceived quality of LGS was assessed with a single item that asked “How would you rate the quality of your local accessible green spaces that are close to your home?” Participants responded from 1 = “Terrible” to 7 = “Excellent”.

NR was assessed using two sections of the NR Scale (NRS; [38]). The self and experience factors were extrapolated to form the NRS-14. The self and experience factor were used to reflect both how strongly people identify with the natural environment and the attraction people have to nature. The perspective factor of the NRS was excluded as we were not interested in global issues such as conservation and species survival rates. Participants were asked to report how they felt about 14 phrases that described their relationship with nature. Examples items included, “Even in the middle of the city, I notice nature around me”, and “I am not separate from nature, but part of nature”. Participants responded using a Likert scale format ranging from 1 = “disagree strongly” to 5 = “agree strongly”. Where appropriate, responses were reversed so that higher scores indicated a greater NR. NR was recorded as a mean of 14 items.

Visit frequency to LGS was assessed by asking participants “How often do you visit the green space closest to your home?” This was rated from 1 = “Every day” to 7 = “Never visit my LGS or any other green spaces”. This score was then reversed scored so that a higher frequency of visits was represented by a higher numerical value. Participants also indicated via multiple choice selection how they usually travelled to LGS, and how long it usually took them.

Self-reported PA levels were recorded using a short-form version of the International Physical Activity Questionnaire (IPAQ-SF, [39]). Participants were required to indicate how many days they undertook PA activity for more than 10 min. Subdomains were vigorous, moderate and walking. Furthermore, participants reported how many hours and minutes they usually spent on these activities on one of those days. Additionally, participants reported how many hours and minutes they would usually spend sitting on a week day.

Raw data were converted into weekly PA levels using IPAQ-SF scoring guidelines [40]. The raw data were calculated into a weekly score described as multiples of the resting metabolic rate (METs). As recommended by IPAQ scoring guidelines, some of the raw data was truncated to reduce potential outliers. Above 180 min in all categories is considered to be unlikely, suggesting participants’ misinterpreted the question. In accordance with guidelines [40], therefore, all moderate minutes that were between 180 and 299 were reduced to 180; those above 299 were divided by seven. Also, vigorous minutes over 180 were divided by seven and walking minutes over 180 were reduced to 180. For the data analysis, participants were dichotomised according to whether they achieved at least 600 MET.min per week or not. Those participants who achieved below 600 MET.min per week in total were classified as not meeting the current minimum requirements for a healthy lifestyle (in accordance with [41]) and in the low category using IPAQ scoring guidelines [40].

A number of variables were included in the study as covariates: age, subjective health, gender, road coverage, environmental deprivation, and active travel to both work and LGS. Environmental Deprivation (at ward level) was obtained from a database that is available on CRESH.org [42]. In summary, ward level measurements were calculated for a variety of environmental dimensions that impact upon health (air pollution, climate, UV radiation, industrial facilities, and green space). Each ward was given a score from −2 to +3, with +3 indicating most deprived environments. For this study, scores of environmental deprivation were reversed so that the most deprived areas had the lowest score.

Road Coverage was calculated by cross referencing ward codes against general land use database [43] across England^1^ to give the amount of road coverage in each ward. This was converted to a percentage of the total land area in each ward. For both environmental deprivation and road coverage, participants’ home post codes were converted to wards using Geoconvert.

Active travel to work was assessed by asking participants “How do you usually travel to work? Tick all that apply”. Any participant who ticked walk or cycle were classified as active commuters. Active travel to LGS was assessed by asking participants “How do you usually travel to your local green space? Tick all that apply” Any participants who ticked walk or cycle were classified as active travellers to LGS.

All data analysis was carried out using IBM SPSS Statistics 20. Three regression models were run. First, an ordinal regression model was run to determine whether objective (% LGS) and subjective (perceived access, perceived quality, and NR) measures predicted frequency of visits to LGS. Additional demographic, objective, and subjective variables were included as covariates in the model (see Table 1).

Second, a binary logistic regression was run to determine whether objective (% LGS) and subjective (perceived access, perceived quality, and NR) measures predicted the likelihood of meeting current UK PA guidelines. Additional demographic, objective, and subjective variables were included as covariates in the model (see Table 2).

Finally, another binary logistic regression was run to determine if visit frequency to LGS predicted the likelihood of meeting current UK PA guidelines. Age, gender and health were included as covariates in the third model. Nagelkerke R^2^ tests were run to assess how much of the variance in the outcomes could be accounted for by the models. Statistical significance was accepted at *p* < 0.05 throughout the analyses.

## Results

The 1379 urban wards represented in the study had a mean green space coverage of 52.7 % (95 % CI, 51.5 to 53.9). This is nearly 10 % lower than the UK national average of 62.6 %. Furthermore, the wards had a mean road coverage of 10.1 % (95 % CI, 3.7 to 16.6 %) and a mean environmental deprivation score of 0.46 (95 % CI, -0.47 to 1.38).

Overall, participants responded favourably towards perceived access (M = 6.15, SD = 1.14) and perceived quality (M = 5.41, SD = 1.23) of LGS; 90.1 % of participants reported at least ‘somewhat easy’ access to LGS and 76.1 % of participants reported perceived quality of LGS as at least ‘good’. Participants reported a mean NR score of 3.29 (SD = 0.73). This is comparable to NR scores reported in previous literature [44, 45].

In this study, engagement with the natural environment is indicated by visit frequency to LGS. In total, 67.7 % of participants reported visiting LGS at least a ‘few times a month’. Active travel to LGS was reported by 85.6 % of participants and the vast majority reported travel duration to LGS of less than 20 min (86.5 %). Additionally, 18.4 % of participants reported actively commuting to their place of work.

In total, 75.5 % of participants (77.7 % of men and 73.2 % of women) reported meeting the current UK PA guidelines of at least 600 MET.min per week [41]. This is higher than national averages (66 % of men and 56 % of women; [4]). Subsequently, 24.5 % of participants did not complete enough MET.min per week to sustain a healthy lifestyle. Participants obtained the most amount of MET.min through walking (M = 54.7 %).

### What predicts visit frequency to LGS?

An ordinal regression was run to predict visit frequency to LGS based on perceptions and objective measures of LGS (see Table 1). A Nagelkerke R^2^ of 0.226 indicates that the model explained 22.6 % of the variation in visit frequency. After controlling for covariates, NR was the strongest predictor of visit frequency to LGS. An increase in NR was associated with an increase in the odds of visiting LGS more frequently (OR = 2.234, 95 % CI, 1.937 to 2.581). Perceived quality of LGS also significantly predicted visit frequency (OR = 1.537, 95 % CI, 1.388 to 1.704), but perceived access did not.

**Table 1:** Odds ratios of visit frequency to LGS

		^a^OR	^b^ 95 % CI	
			Lower	Upper
Covariates	*Age*	0.994	0.985	1.002
	*Health*	1.071	0.985	1.165
	*Gender*	1.003	0.832	1.210
	*% of Road Coverage*	1.011	0.976	1.047
	*Environmental Deprivation*	1.082	0.970	1.206
	*Active Travel to Work*	1.125	0.915	1.384
Objective	*% of Local Green Space*	1.006	0.998	1.015
Subjective	*Perceived Access*	1.106	0.994	1.230
	*Perceived Quality*	1.537*	1.388	1.704
	*Nature Relatedness*	2.234*	1.937	2.581

Note. R^2^ = .226 (Cox and Snell), .226 (Nagelkerke). Model χ^2^ (10) = 348.022, *p* < 0.01. ^a^Odds Ratios, ^b^95% Confidence Intervals, * indicates significance at *p* < 0.01

### What predicts whether participants meet PA guidelines?

A binary logistic regression was run to determine which variables predicted the likelihood of meeting PA guidelines (see Table 2); the model explained 13.1 % of the variance (Nagelkerke R^2^ = 0.131). After controlling for covariates, NR was the only significant predictor of meeting PA guidelines (OR = 1.268, 95 % CI, 1.128 to 1.424). Neither pLGS (Access and Quality) nor objectively measured green space were significant predictors. Of the covariates, subjective health and active travel to both work and LGS were significant.

**Table 2:** Odds ratios of meeting PA guidelines

		^a^OR	^b^95% CI	
			Lower	Upper
Covariates	*Age*	0.994	0.982	1.006
	*Health*	1.268*	1.128	1.424
	*Gender*	0.779	0.601	1.010
	*% of Road Coverage*	1.017	0.969	1.067
	*Environmental Deprivation*	0.997	0.859	1.158
	*Active Travel to Work*	1.971*	1.441	2.695
	*Active Travel to Local Green Space*	1.600*	1.076	2.378
Objective	*% of Local Green Space*	0.994	0.982	1.006
Subjective	*Perceived Access*	0.993	0.856	1.151
	*Perceived Quality*	1.042	0.908	1.197
	*Nature Relatedness*	1.268*	1.128	1.424

Note. R^2^ = .089 (Cox and Snell), .131 (Nagelkerke). Model χ^2^ (11) = 125.680, *p* < 0.01. ^a^Odds Ratios, ^b^95% Confidence Intervals, * indicates significance at *p* < 0.01

### Does visit frequency to LGS predict whether participants meet PA guidelines?

**Fig. 1:**
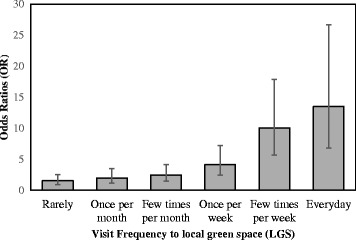
Odds ratios of achieving physical activity guidelines by visit frequency to local green space (compared to never going). The odds ratios (OR) and 95 % Confidence Intervals of meeting physical activity guidelines (600 MET.min per week), compared with never going to LGS

A binary logistic regression was run to predict the likelihood of meeting PA guidelines based upon visit frequency to LGS. The model explained 16.8 % of the variation in whether participants met PA guidelines (Nagelkerke R^2^ = 0.168). As illustrated by Fig. 1, as visit frequency to LGS increased so did the likelihood of achieving PA guidelines (compared to never going).

## Discussion

This study found that as the number of visits to LGS increased so did the odds ratio of meeting PA guidelines. The findings also highlight the importance of NR, which was the strongest predictor of both visit frequency to LGS and meeting PA guidelines. In contrast, objectively measured quantity of LGS was not a significant predictor of visit frequency to LGS or meeting PA guidelines. Furthermore, perceived quality and perceived access did not significantly predict the likelihood of meeting PA guidelines, but perceived quality of LGS did significantly predict visit frequency.

The current research was the first nationwide study to examine the relationship between LGS, visits to LGS, and PA in the UK. Previous studies (see [10] for a review) have often investigated visits to all natural or green spaces regardless of proximity to home. This study specifically asked respondents about LGS closest to home. LGS are places that are close to homes and therefore should be accessible for the majority irrespective of whether the household owns a car.

Furthermore, the current study was the first study to assess NR, pLGS, and objectively measured LGS as predictors of visit frequency to LGS and PA levels. Not only does this expand upon the existing literature regarding objectively measured LGS and PA, the findings highlight the importance of subjective variables relating to LGS.

Within this study we examined the influence of subjective measures associated with LGS on green space usage. Consistent with previous research [30] we found that NR was positively associated with visit frequency to LGS. In fact, over and above a variety of independent variables and covariates, NR was the strongest predictor of visit frequency to LGS. In doing so, our evidence supports the schematic model proposed by Calogiuri et al. [10], in which feelings about nature are related to intentions to visit LGS. It also supports the notion that visit frequency to LGS moderates the relationship between NR and psychological well-being (as highlighted by [31]). Green exercise research suggests that PA in LGS can have a positive effect on many indices associated with psychological well-being [46]. Therefore, visiting LGS more often is likely to increase psychological well-being and further investigation is warranted to assess what role NR has on this relationship.

The current findings suggest that pLGS may impact upon behaviour more than quantity of LGS. In addition to the influence of NR, perceived quality also significantly predicted visit frequency to LGS but objectively measured quantity of LGS did not. With regards to perceived quality of LGS, previous research may give an indication of how this could be enhanced, with perceived attractiveness, perceived availability of features [47] and park characteristics [48] all suggested to play an important role in the relationship between LGS and PA.

Neither perceived access nor perceived quality of LGS significantly predicted whether participants met PA guidelines. One possible explanation for this is the high percentage (75.5 %) of participants who met PA guidelines (14.5 % higher than the national average in England). In fact, males were 11.7 % and females were 17.2 % higher than the national average. This is most likely due to the sample being exclusively employed people. Current evidence suggests that those in formal employment were more likely to know the current recommendations for PA in the UK, and be physically active [49].

One of the main strengths of this study is the inclusion of both subjective and objective measures of LGS. Most previous studies in this area compared quantity of LGS (described as objectively measured quantity or perceived access) with PA. Our study added more robustness to this relationship with additional subjective measures. We expanded the limited research on NR and have shown its importance in the relationship between LGS and PA.

Against these contributions, some limitations should be noted. First, due to the correlational nature of the study, causality cannot be inferred in the observed relationships. Second, the study used a self-reported measure of PA. Although the IPAQ is well used in the literature, people often over-estimate PA levels [50]. Furthermore, this study did not explore PA in detail. Had we also explored ‘green exercise’, as opposed to just overall PA levels, we may have been able to provide stronger explanations for the results. Further investigation of green exercise, distinct from PA, is warranted to provide better understanding of the mechanisms between LGS, PA and health.

One further limitation is the double inclusion of objectively measured LGS: the environmental deprivation score - that was used as a covariate - was calculated in part using objectively measured LGS. This was deemed necessary as it included a variety of additional factors such as climate, and pollution etc..^2^ Although efforts were made to account for environmental factors, the level of detail required to accurately portray the favourableness of home location for green exercise was beyond the reach of this study. For example, street lighting and pedestrian pathways that link housing areas to LGS may influence visit frequency.

As mentioned previously, the inclusion of only employed individuals does limit the ability to generalise the findings to other populations e.g. unemployed, retired. Likewise, whilst active commuters were controlled for in statistical analysis, this analysis did not explore visiting green space during work hours, and the subsequent impact this may have had on PA levels. Additionally, we did not account for variations in employment type. Further work is needed to explore how the complexities of working life (location, activity levels, environment etc.) influence the relationships we found.

Results from this study show that on average participants had less LGS than the national average at ward level. Even though the percentage of employed people is about the same for rural and urban areas in England, the vast majority of people in England live in urban areas (81.5 % of people in 2011). We suggest that the inclusion of only employed participants skewed the results towards more urbanised wards. It is therefore likely that the majority of participants reported visits to urban green space rather than natural environments, although we do not have the data to confirm this.

## Conclusion

This is the first nationwide study to explore the relationship between LGS and PA. We found that visit frequency to LGS is associated with the likelihood of meeting PA guidelines. Furthermore, subjective measures of LGS, and particularly NR, appear to be more important than objectively measured quantity of LGS for predicting both visit frequency to LGS and PA. As PA is known to have many positive health benefits, visits to LGS, especially if active transport is used, potentially could have a significant impact on Public Health.

## Availability of data and materials

The dataset supporting the conclusions of this article is available in the UK Data Service repository http://reshare.ukdataservice.ac.uk/852253/.

## Abbreviations

LGS: local green space
MET.min: MET minutes
METs: multiples of the resting metabolic rate
NR: nature relatedness
PA: physical activity
pLGS: perceptions of local green space

## Footnotes

1 Road coverage for Scotland, Wales, and Northern Ireland was recorded as missing data and treated as such in the analyses. The analyses were re-run including only participants from England and the same pattern of results was observed. As such, results from the full sample are reported in the manuscript.

2 Statistical analysis revealed that when environmental deprivation was removed as a covariate, objectively measure quantity of LGS was not a significant predictor of visit frequency to LGS or PA. Therefore, environmental deprivation – as a covariate – was included in the main analysis.
